# Minimally Invasive Lumbar Interbody Fusion With an Expandable Meshed Allograft Containment Device: Analysis of Subsidence With 12-Month Minimum Follow-Up

**DOI:** 10.14444/6044

**Published:** 2019-08-31

**Authors:** JOHN PAUL G. KOLCUN, GEORGE M. GHOBRIAL, KENNETH M. CRANDALL, KEN HSUAN-KAN CHANG, GIACOMO PACCHIAROTTI, MICHAEL Y. WANG

**Affiliations:** 1 Department of Neurological Surgery, University of Miami Miller School of Medicine, Miami, Florida; 2 Department of Neurological Surgery, National Yang-Ming University School of Medicine, Taipei, Taiwan; 3 Institute of Neurosurgery, University of Rome “La Sapienza,” Rome, Italy

**Keywords:** lumbar interbody fusion, transforaminal lumbar interbody fusion, minimally invasive surgery, interbody device, subside

## Abstract

**Background:**

We have previously reported the use of a minimally invasive allograft-filled expandable meshed-bag containment system in the lumbar spine. Subsidence has not been reported with this device. In this retrospective case series, we describe subsidence after lumbar interbody fusion using this device, with 12-month minimum radiographic follow-up.

**Methods:**

Consecutive adult patients that underwent 1- or 2-level interbody fusion with at least 1 year of follow-up were included in this study. Preoperative, postoperative, and final follow-up lumbar radiographs were analyzed to measure disc height at the anterior and posterior margins of the disc space, as well as the neuroforaminal height.

**Results:**

Forty-one patients were identified, with a mean age of 63.4 years (± 11.8). A total of 61 levels were treated, with successful fusion observed in 54 levels (88.5%). The mean radiographic follow-up was 24.3 months (± 11.2). The mean disc height pre- and postoperatively was 6.9 mm (± 3.2) and 10.1 mm (± 2.9, *P* < .001), respectively. The mean disc height at final follow-up was 8.3 mm (± 2.4). Average disc height subsidence was 1.8 mm (± 1.7, *P* < .001). Overall, average disc height increased by a net 1.3 mm (± 2.5, *P* < .001). The mean neuroforaminal height pre- and postoperatively was 18.0 mm (± 3.3) and 20.7 mm (± 3.6, *P* < .001), respectively. The mean neuroforaminal height at final follow-up was 19.2 mm (± 3.4). Average neuroforaminal height subsidence was 1.3 mm (± 3.4, *P* = .012). Overall, average neuroforaminal height increased by a net 1.7 mm (± 2.8, *P* = .004). No significant difference in subsidence was observed between 1- and 2-level surgeries.

**Conclusion:**

An expandable allograft containment system is a feasible alternative for lumbar interbody fusion. Due to its biologic and mechanical nature, the surgeon using such constructs should account for an anticipated average of 18% loss of interbody height due to subsidence during the bony remodeling/fusion process.

## INTRODUCTION

As populations age, the annual rate of lumbar fusion procedures performed in developed nations is steadily increasing.^1^ Open lumbar interbody fusion procedures can carry a higher morbidity in elderly patients due to increased comorbidities and impaired cardiopulmonary reserves. These patients also tend to have a longer postoperative recovery period.^2^ The current drive to decrease surgical invasiveness while achieving the same operative outcomes benefits fragile patient populations, such as the elderly. Minimally invasive surgery (MIS) embodies the attempt to decrease surgical morbidity, postoperative pain, and the postoperative hospital recovery period.^3^ Compared to the traditional open approach, MIS transforaminal lumbar interbody fusion (TLIF) has been associated with reduced blood loss and tissue destruction, earlier mobilization, and shorter hospital stays.^4^ While the traditional open TLIF approach offers a larger working channel for endplate preparation, fusion rates between open and MIS TLIF are generally comparable.^5^ Subsidence greater than 3 mm is generally considered significant, and acceptable rates of subsidence have been reported with both open and MIS techniques.^6^,^7^

The OptiMesh device (Spineology, Saint Paul, Minnesota) is a small, expandable polyethylene meshed sac, originally designed to contain allograft within a vertebral body defect. However, the OptiMesh can also be placed within the interbody space with reduced approach-related tissue destruction, while still achieving sufficient endplate preparation. With this method, fusion rates comparable to other interbody devices may be attainable. Over time, however, excessive graft subsidence could potentially restrict the neuroforamen, leading to a recurrence of symptoms.

The authors report the use of an OptiMesh expandable meshed allograft containment system for 1- or 2-level lumbar degenerative indications, with a minimum 12-month radiographic follow-up.

## MATERIALS AND METHODS

### Data Collection

Consecutive records in a single-surgeon database (M.Y.W.) were reviewed for patients greater than 18 years old who underwent interbody fusion with the OptiMesh device at a single institution from August 2009 to October 2015. It should be noted that this application of the cage is designated as “off-label” by the US Food and Drug Administration. Patients included in this study were treated for 1- or 2-level lumbar disease with a minimum of 12 months of follow-up. Demographic, clinical, and operative information was collected for each patient, including age, sex, tobacco use, indication for surgery, operative level, estimated blood loss, fusion rate, postoperative hospital stay, complications, and length of follow-up.

### Surgical Technique

The technique used to place this device in the lumbar interbody space has been reported previously.^8^ Height restoration is achieved with increasing allograft placement in the graft containment system, which grows to fill and expand the interbody space. Recombinant human bone morphogenetic protein-2 (rhBMP-2, Infuse, Medtronic, Minneapolis, Minnesota) at a dose of up to 1.05 mg/level is utilized prior to mesh graft placement (within the interbody space but outside the cage).

### Radiographic Measurements

Anteroposterior (AP), lateral, and dynamic flexion/extension x-ray images for each patient were reviewed and measured. The lateral lumbar x-ray was reviewed for disc height, neuroforaminal height, anterolisthesis, lumbar lordosis, and interbody angle.^9^ Lumbar Cobb angle was measured from AP lumbar views. Computed tomography (CT) images and dynamic x-rays were reviewed to assess fusion (Figure 2).

**Figure 1:**
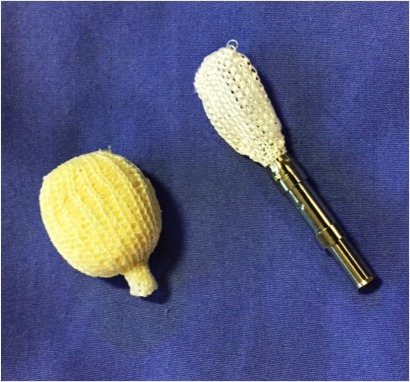
Model of the OptiMesh expandable mesh allograft containment device, shown empty (loaded onto applicator) and expanded (filled with allograft).

**Figure 2:**
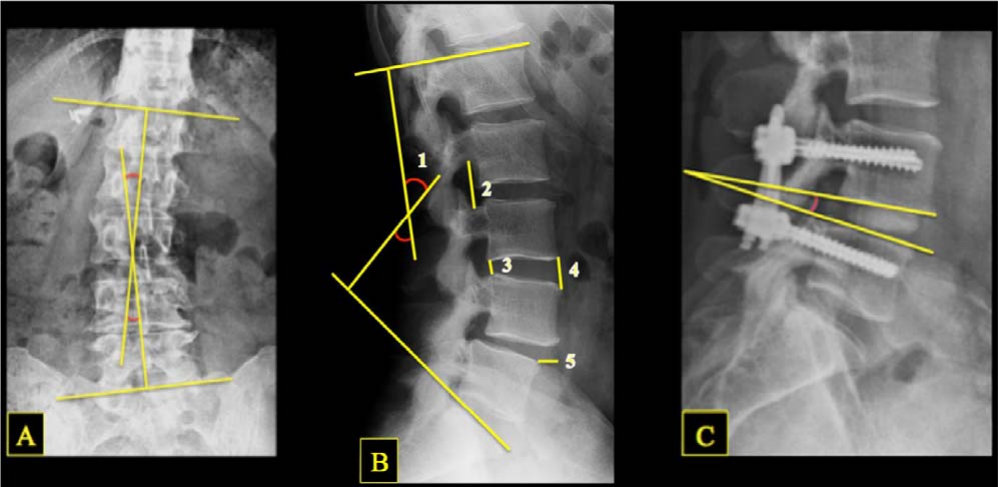
Illustration of x-ray measurements, showing lumbar Cobb angle (A), lumbar lordosis (B1), neuroforamen height (B2), posterior disc height (B3), anterior disc height (B4), anterolisthesis (B5), and postoperative interbody angle (C).

**Figure 3:**
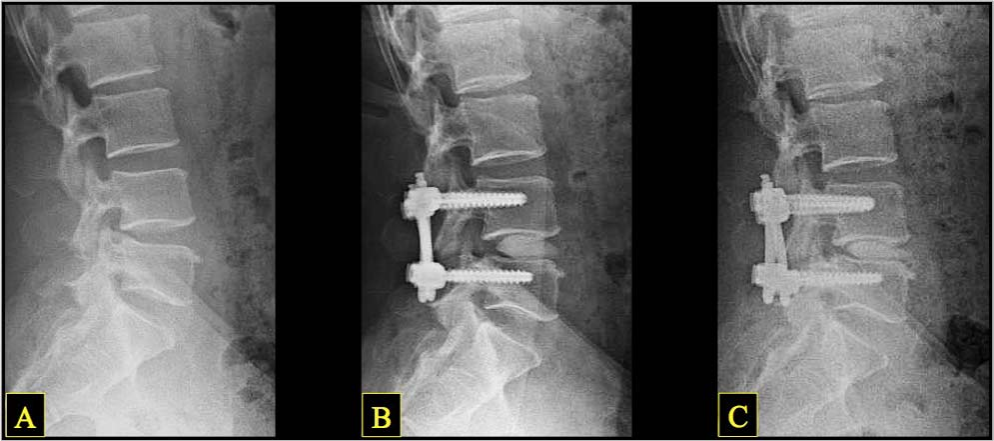
Representative lateral lumbar x-ray sequence in a single patient, showing preoperative (A), postoperative (B), and long-term follow-up (C) images. Note the changes in disc and neuroforaminal height: a marked increase from A to B, and slight decrease from B to C.

Subsidence was defined as a loss of average disc height and/or neuroforaminal height between postoperative and final images. Successful postoperative fusion was defined as: (1) continuous bridging bone observed on CT scan or, if CT was unavailable or inconclusive, (2) < 3° of motion and < 3 mm of translation observed on dynamic lumbar lateral x-ray (flexion/extension), with no evidence of screw dislodgment, migration, or screw-rod breakage^10^ (Figure 3).

### Statistical Analysis

Continuous variables were analyzed with 2-tailed paired Student *t* tests (pre- versus postoperative) and 2-tailed independent Welch *t*-tests (1 versus 2 levels). Categorical variables were analyzed with a Pearson χ^2^ test. A *P* value of < .05 was considered statistically significant. Continuous variables are reported as mean (± SD). Categorical variables are reported as n (%). Statistical calculations were performed in Microsoft Excel 2011 (Microsoft, Redmond, Washington).

## RESULTS

### Population Characteristics

Forty-one patients were identified, with a mean age of 63.4 years (± 11.8). A majority of patients were female (29, 70.7%). A total of 61 spinal levels were treated, with a majority of procedures performed at the L4-5 level (29, 70.7%). Roughly one-quarter of patients reported tobacco use (11, 26.8%). The most common indication for surgery was spondylolisthesis (22, 53.7%). The average blood loss was estimated at 203.9 mL (± 186.5). Fusion was observed in 54 levels (88.5%) by the 12-month interim. Patients spent an average 4.3 days (± 2.3) in the hospital postoperatively. One complication was observed (2.4%): a fractured rod, which occurred in a patient with pseudoarthrosis. The mean radiographic follow-up was 24.3 months (± 11.2) (Table 1).

**Table 1:** Baseline population characteristics, indications, and outcomes. Continuous variables are represented as mean ± SD, and categorical variables as n (%).

Parameter	Value
Demographics	
No. of patients	41
Age (years)	63.4 ± 11.8
Sex ratio (M:F)	12 (29.3):29 (70.7)
Tobacco use	11 (26.8)
Operative level	
L2-3	2 (4.9)
L3-4	15 (36.6)
L4-5	29 (70.7)
L5-S1	15 (36.6)
Outcomes	
Fusion (levels)	54 (88.5)
EBL (mL)	203.9 ± 186.5
LOS (days)	4.3 ± 2.3
Complications	1 (2.4)
Follow-up (months)	
Clinical	28.4 ± 13.5
Radiographic	24.3 ± 11.2

Abbreviations: EBL, estimated blood loss; LOS, length of hospital stay.

### Radiographic Outcomes

**Figure 4:**
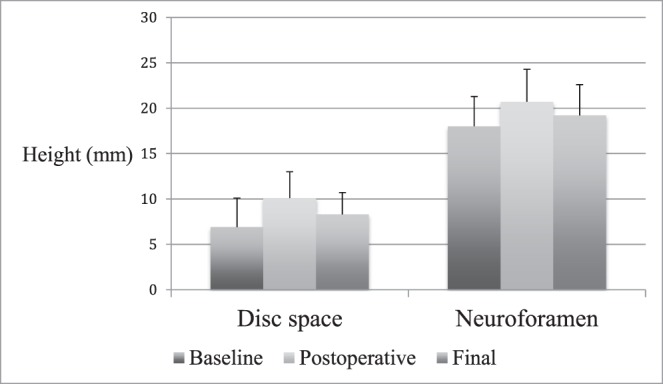
Total population radiographic findings. Disc space and neuroforamen height (both in mm) shown at baseline, postoperative, and final follow-up.

Postoperatively, disc height increased by 3.1 mm (± 1.9, 66.5%, *P* < .001) and neuroforaminal height increased by 3.0 mm (± 3.3, 19.1%, *P* < .001). At final follow-up, the disc height had decreased by 1.8 mm (± 1.7, 15.5%, *P* < .001) and neuroforaminal height had decreased by 1.3 mm (± 3.4, 4.3%, *P* = .012) from postoperative levels. Interbody angle decreased by 0.8° (± 2.9, 5.2%, *P* = .033) between postoperative and final imaging. Neuroforaminal height change was significantly less than that of the average disc height (4.3% versus 15.5%, *P* < .001). From baseline to final radiographic follow-up, disc height increased by a net 1.3 mm (± 2.5, 42.8%, *P* < .001) and neuroforaminal height increased by a net 1.7 mm (±2.8, 11.2%, *P* = .004) (Figure 4).

Similar results were seen in a net reduction of anterolisthesis by 1.3 mm (± 2.6, 29.9%, *P* = .004) and lumbar Cobb angle by 1.1° (± 4.3, 20.5%, *P* = .078), and a net increase in lumbar lordosis by 0.3° (± 11.9, 14.4%, *P* = .010). While less robust, these effects were significant (Table 2).

**Table 2:** Total sample radiographic measurements at baseline, postoperatively, and at most recent follow-up. All values are represented as mean ± SD. All lengths are given in millimeters, and all angles shown in degrees. Comparisons between time points are shown with results of 2-tailed paired Student t tests.

	Disc Height			Foraminal Height	Anterolisthesis	Lumbar Lordosis	Lumbar Cobb Angle	Interbody Angle
	Anterior	Posterior	Average					
Baseline	9.2 ± 4.4	5.1 ± 2.2	6.9 ± 3.2	18.0 ± 3.3	3.7 ± 3.8	40.3 ± 14.8	5.4 ± 8.2	...
Postoperative	12.8 ± 3.1	8.2 ± 2.3	10.1 ± 2.9	20.7 ± 3.6	2.2 ± 3.0	42.9 ± 12.7	4.0 ± 6.4	6.7 ± 4.3
Change from baseline	3.3 ± 2.7	2.9 ± 2.2	3.1 ± 1.9	3.0 ± 3.3	−1.3 ± 2.6	0.5 ± 12.5	−1.1 ± 4.3	...
Change from baseline, %	56.9	87.2	66.5	19.1	−29.9	29.8	−14.7	...
* P* value	< .001	< .001	< .001	< .001	.004	.018	.065	
Final	10.8 ± 2.5	6.6 ± 2.0	8.3 ± 2.4	19.2 ± 3.4	...	42.7 ± 13.9	4.2 ± 6.6	5.9 ± 4.4
Change from postop	−2.0 ± 2.0	−1.5 ± 1.8	−1.8 ± 1.7	−1.3 ± 3.4	...	−0.3 ± 21.3	0.2 ± 2.3	−0.8 ± 2.9
Change from postop, %	−15.0	−17.1	−15.5	−4.3	...	−0.4	26.4	−5.2
* P* value	< .001	< .001	< .001	.012		.689	.536	.034
Net changes								
Change from baseline	1.3 ± 3.4	1.4 ± 2.6	1.3 ± 2.5	1.7 ± 2.8	−1.3 ± 2.6	0.3 ± 11.9	−1.1 ± 4.3	−0.8 ± 2.9
Change from baseline, %	34.6	57.9	42.8	11.2	−29.9	14.4	−20.5	−5.2
* P* value	.062	.002	< .001	.004	.004	.010	.078	.034

**Figure 5:**
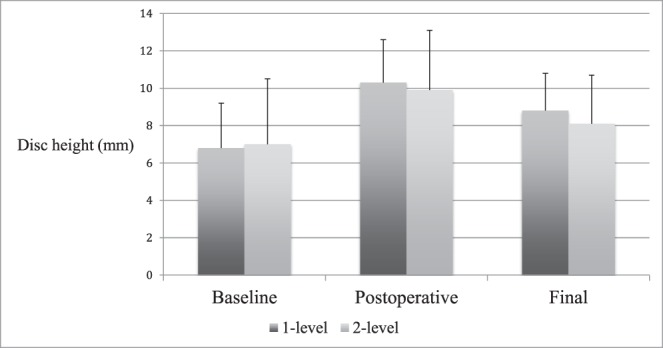
Subgroup radiographic findings. Average disc height (mm) shown at baseline, postoperative, and final follow-up. There were no significant differences in disc height or subsidence between 1- and 2-level patients.

**Figure 6:**
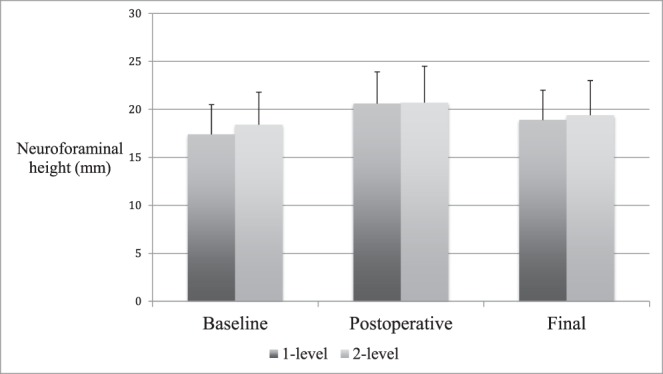
Subgroup radiographic findings. Neuroforamen height (mm) shown at baseline, postoperative, and final follow-up. There were no significant differences in neuroforaminal height or subsidence between 1- and 2-level patients.

Radiographic parameters in patients undergoing 1- or 2-level surgery were compared. Differences in demographics were assessed to justify comparing these groups. A greater number of 2-level patients reported tobacco use (*P* = .010). Total follow-up period was longer in 2-level patients, (*P* = .019), although in both groups the follow-up was at minimum 12 months. Generally, radiographic changes were comparable between groups. However, 1-level patients did experience a loss of lumbar lordosis between postoperative and final imaging, while lordosis increased slightly in 2-level patients (−10.6% versus +5.3%, *P* = .050). This effect bordered on significance. Net change in lumbar lordosis was not significantly different between groups (*P* = .327). Further, 1-level patients tended toward a greater reduction in interbody angle between postoperative and final imaging. This effect approached significance (*P* = .082) (Tables 3 and 4; Figures 5 and 6).

**Table 3:** Subgroup population characteristics, indications, and outcomes. Continuous variables are represented as mean ± SD, and categorical variables as n (%). Comparisons shown as results of 2-tailed Welch t test (continuous variables) and Pearson χ^2^ test (categorical variables). P-values achieving statistical significance are shown in bold.

	1 Level	2 Levels	*P* Value
Demographics			
No. of patients	21	20	
Age (years)	62.2 ± 12.0	64.6 ± 11.8	.539
Sex ratio (M:F)	7 (33.3):14 (66.7)	5 (25.0):15 (75.0)	.558
Tobacco use	2 (9.5)	9 (45.0)	**.010**
Operative level			
L2-3	1 (4.8)	1 (2.5)	.637
L3-4	2 (9.5)	13 (32.5)	**.048**
L4-5	10 (47.6)	19 (47.5)	.993
L5-S1	8 (38.1)	7 (17.5)	.076
Outcomes			
Fusion (levels)	18 (85.7)	34 (85.0)	.940
EBL (mL)	205.3 ± 236.2	202.6 ± 125.2	.966
LOS (days)	4.1 ± 2.7	4.7 ± 1.6	.397
Complications	1 (2.4)	0 (0)	.323
Follow-up (months)			
Clinical	26.9 ± 16.4	30.0 ± 9.7	.473
Radiographic	20.2 ± 7.3	28.4 ± 12.9	**.019**

Abbreviations: EBL, estimated blood loss; LOS, length of hospital stay.

**Table 4:** Subgroup radiographic measurements at baseline, postoperatively, and at most recent follow-up. All values are represented as mean ± SD. All lengths are given in millimeters, and all angles shown in degrees. Comparisons of percentage of change at each point are shown with results of 2-tailed Welch t tests.

	Disc Height			Foraminal Height	Anterolisthesis	Lumbar Lordsosis	Lumbar Cobb Angle	Interbody Angle
	Anterior	Posterior	Average					
Baseline								
1 level	9.4 ± 4.1	5.0 ± 1.7	6.8 ± 2.4	17.4 ± 3.1	3.0 ± 4.1	41.6 ± 14.5	3.1 ± 5.1	...
2 levels	9.1 ± 4.6	5.2 ± 2.5	7.0 ± 3.5	18.4 ± 3.4	4.0 ± 3.6	38.2 ± 13.7	7.9 ± 10.2	...
Postop								
1 level	13.4 ± 3.0	7.9 ± 2.0	10.3 ± 2.3	20.6 ± 3.3	1.5 ± 2.5	47.0 ± 10.9	2.1 ± 3.8	7.3 ± 4.8
Change from baseline	3.8 ± 2.4	3.2 ± 1.8	3.5 ± 1.7	3.5 ± 1.5	1.1 ± 2.7	5.4 ± 7.8	−1.0 ± 2.7	...
Change from baseline, %	65.9	95.3	70.3	20.5	27.4	66.3	−10.7	...
2 levels	12.4 ± 3.1	8.4 ± 2.5	9.9 ± 3.2	20.7 ± 3.8	2.5 ± 3.2	38.7 ± 13.2	6.0 ± 8.0	6.4 ± 4.1
Change from baseline	3.1 ± 2.9	2.7 ± 2.4	2.9 ± 2.0	2.8 ± 3.9	1.4 ± 2.6	0.5 ± 7.0	−2.0 ± 6.6	...
Change from baseline, %	51.9	82.4	64.4	18.4	30.9	4.5	−11.3	...
* P* value	.491	.722	.799	.682	.834	.250	.989	
Final								
1 level	12.0 ± 2.6	6.4 ± 1.6	8.8 ± 2.0	18.9 ± 3.1	...	45.3 ± 14.1	2.3 ± 4.1	5.8 ± 4.3
Change from postop	−2.0 ± 2.6	−1.8 ± 2.0	−1.9 ± 2.2	−2.4 ± 5.0	...	−3.8 ± 9.1	−0.1 ± 2.1	−1.9 ± 3.2
Change from postop, %	−15.8	−20.7	−17.9	−10.4	...	−10.6	−1.6	−21.9
2 levels	10.1 ± 2.2	6.8 ± 2.3	8.1 ± 2.6	19.4 ± 3.6	...	40.1 ± 13.6	6.1 ± 8.1	6.0 ± 4.5
Change from postop	−2.3 ± 2.2	−1.5 ± 1.9	−1.9 ± 1.8	−1.3 ± 4.1	...	1.4 ± 5.2	0.2 ± 3.4	−0.4 ± 2.7
Change from postop, %	−16.7	−17.3	−16.3	−3.5	...	5.3	26.1	−0.7
* P* value	.895	.628	.791	.256		.050	.447	.082
Net changes								
1 level								
Change from baseline	2.3 ± 3.4	1.7 ± 2.5	1.6 ± 3.0	2.0 ± 1.6	1.1 ± 2.7	3.8 ± 5.6	−1.1 ± 2.2	−1.9 ± 3.2
Change from baseline, %	52.9	70.0	49.1	12.0	27.4	30.5	−30.1	−21.9
2 levels								
Change from baseline	0.8 ± 3.3	1.2 ± 2.6	1.0 ± 2.4	1.5 ± 3.2	1.4 ± 2.6	1.9 ± 7.4	−1.8 ± 6.6	−0.4 ± 2.7
Change from baseline, %	23.6	49.2	35.5	10.5	30.9	8.2	−5.4	−0.7
* * *P* value	.270	.623	.661	.707	.834	.327	.239	.082

## DISCUSSION

In this retrospective study using an expandable meshed allograft-containment interbody device, we find that rates of fusion and graft subsidence at a minimum 12 months postoperatively are not inferior to those reported with conventional interbody devices.

No prior reports describe subsidence rates for the OptiMesh system in patients with 1- and 2-level lumbar degenerative disc disease. This information is of particular importance while measuring the disc space height preoperatively to determine graft height selection,^11^ as oversizing of interbody grafts has been implicated as the single most important technique-related risk for subsidence.^12^

MIS techniques are designed to reduce approach-related soft tissue trauma and operative duration with the goal of delivering equivalent outcomes with a shorter recovery time.^3^ However, the adoption and promotion of new surgical technology must be predicated on demonstrated, long-term efficacy. Cadaveric studies have demonstrated the relationship between disc height, neuroforamen height, and nerve compression, as well as the biomechanical capability of the OptiMesh expandable device to manipulate this anatomy effectively.^13^,^14^ Numerous reports have shown the durability of expandable technologies in providing symptomatic relief via indirect decompression.^9^,^11^,^15^,^16^ We have previously reported our use of the OptiMesh device in 25 patients with adult degenerative scoliosis after 1 year of follow-up, demonstrating no recurrent radiculopathic symptoms along the concavity.^8^ In this study, significant improvements were achieved in the Cobb angle (20.2° reduction), lumbar lordosis (14.8° increase), and global sagittal alignment (3.1-cm reduction). Improvements were also seen in various clinical parameters, including the numeric pain scale extremity score (3.3-point reduction), numeric pain scale axial back score (4.2-point reduction), and Oswestry disability index (20.8-point reduction).^8^

A number of methods for evaluating postoperative fusion and subsidence have been described previously. Early methods for assessing segmental fusion relied on standing radiographs, which could be used to observe bridging bone through the fusion mass or segmental motion during flexion or extension.^17^,^18^ Later studies determined that CT imaging is a more accurate and reliable measure of the presence and quality of fusion.^19^,^20^ Consequently, we utilized CT images whenever possible to assess fusion in our patients. Observation of fusion bridging through the OptiMesh graft is a particular challenge due to the homogenous hyperdense radiographic appearance of the graft in the interbody space, likely the result of impacting morcelized allograft into the fixed-volume mesh bag. Therefore, traditional methods of observing bridging fusion through the structural graft via the cancellous portion were not always feasible in our patient sample.^17^ Instead, flexion-extension imaging—a reliable method for determining the absence of fusion—could be used to assess fusion in this subset of patients, as previously described.^10^

Previous studies have reported subsidence rates with other expandable implants. Loss of interbody height with expandable polyaryl-ether-ether-ketone (PEEK) spacers has been reported from 0.6 to 1.1 mm in patients with follow-up comparable to the present study, and higher (2 to 4 mm) with longer follow-up.^9^,^11^,^21^ Isaacs et al compared outcomes utilizing PEEK spacers placed either by MIS lateral interbody fusion or by MIS TLIF.^22^ They found greater subsidence with MIS TLIF as compared to the lateral approach, which was thought to be due to the larger graft footprint and the ability to contact the denser apophyseal ring. However, both MIS and lateral lumbar interbody fusion subsidence rates were comparable with findings in previous literature regarding PEEK spacers, as well as the present study of the OptiMesh device (1.3 mm with MIS TLIF versus 0.8 mm with lateral lumbar interbody fusion). Only one prior study reported neuroforaminal subsidence, with an average loss of 1.1 mm height at 12 months.^11^ This study failed to achieve significant postoperative improvement in the neuroforaminal height over the full course of follow-up.

Our results suggest that the OptiMesh system offers an acceptable subsidence rate at 1 to 2 years postoperatively, at levels similar to previous reports. However, variation in the use and dosage of rhBMP-2 may influence subsidence, precluding a strong, direct comparison between studies.^12^,^23^ The use of rhBMP-2 has been associated with both accelerated postoperative fusion^24^ and an increased rate of subsidence,^25^,^26^ among other complications. To date, the optimal dosage and indications for rhBMP-2 have yet to be rigorously defined.^23^

The use of the OptiMesh graft allows safe access to the disc space with minimal tissue destruction, as demonstrated by the absence of durotomy, symptomatic nerve injuries, and wound-healing complications, all of which have been reported with open TLIF and posterior lumbar interbody fusion procedures.^27^ Further, there was no significant difference in subsidence rates between patients treated at either 1 level or 2 consecutive levels. These results are promising for future study of maintenance of clinical outcomes with the OptiMesh expandable device to further evaluate the durability of this interbody device.

Limitations of this study include the retrospective design, lack of clinical outcomes measures, and follow-up limited to 1 year. Prospective study of the interbody system with an emphasis on radiographic and clinical endpoints at regular postoperative intervals would be the ideal method to determine precisely the incidence, rate, and clinical relevance of subsidence with the OptiMesh expandable device.

## CONCLUSION

The use of the OptiMesh graft containment device for 1- or 2-level lumbar interbody fusion is feasible, with a high fusion rate at 12 months. While a low incidence and degree of subsidence was observed, further study in a prospective fashion would better characterize these endpoints and their clinical significance.
